# Divining the Essence of Symbiosis: Insights from the Squid-Vibrio Model

**DOI:** 10.1371/journal.pbio.1001783

**Published:** 2014-02-04

**Authors:** Margaret McFall-Ngai

**Affiliations:** Department of Medical Microbiology and Immunology, University of Wisconsin-Madison, Madison, Wisconsin, United States of America

## Abstract

Recent molecular data place microbes at the center of the biosphere, from ecosystem sustainability to animal and plant fitness. Models, including the squid-vibrio symbiosis described in this Essay, provide windows into underlying mechanisms that drive these systems.

## Abstract

Biology has a big elephant in the room. Researchers are learning that microorganisms are critical for every aspect of the biosphere's health. Even at the scale of our own bodies, we are discovering the unexpected necessity and daunting complexity of our microbial partners. How can we gain an understanding of the form and function of these “ecosystems” that are an individual animal? This essay explores how development of experimental model systems reveals basic principles that underpin the essence of symbiosis and, more specifically, how one symbiosis, the squid-vibrio association, provides insight into the persistent microbial colonization of animal epithelial surfaces.

## The Broader Context: Why Symbiosis, Why Model Systems?

The field of biology has reached an inflection point, enabled by advances in nucleic-acid sequencing technology. New knowledge of the diversity and centrality of the microbial world promises to change the face of this discipline, shaking its very foundations [1]. As Eugene Koonin points out in his new book, *The Logic of Chance* [2], this recent recognition of the impact of microbes, coupled with other advances in the theory of evolution, will result in a serious, 21st-century reformulation of our basic concepts into a “Post-Modern Synthesis.” The integration of three features of current-day biology promise to drive this new framework: the molecular revolution, which began in the 1950s; the advent over the last decade of broad-scale comparative genomics; and the recognition of microbes, most of which do not have the vertical transmission of hereditary material typical of animals and plants, as the dominant life forms. This “Post-Modern Synthesis” will go far beyond the “Modern Synthesis,” which was the powerful framework that integrated the work of Darwin and Mendel, and unified biology in the last century.

As a result of these advances, one area of intense and growing interest in recent years has been the association of microbes with animals [3], particularly humans (for review see [4]), where 90% of the cells are now known to be bacterial. The normal function of this ecosystem undergoes a classical succession from birth to death, a progression that is critical for such disparate processes as brain development and resistance to various pathologies, including cancer, obesity, and autoimmune diseases. While mammals are the best-studied systems at this time, it is widely believed that all animals and plants form similarly essential partnerships with bacteria and other microbes.

How will we define the basic “rules” governing symbiotic associations? To tackle other complex problems, biologists have turned to studying simpler models. Their exploitation provides insight in two questions: (1) What features (cellular, molecular, biochemical) are conserved through evolution, even in the face of the varying form and function of animals? The principle at work here is that features invariant over geological time are likely to be fundamental and critical. (2) What elements are under selection to create diversity, or how can a basic process be performed differently? The principle here is that, while the system retains a certain core nature (here, an animal-bacterial interaction), specializations for particular functions occur within each of the partners. Both of these aspects inform us about the essence of the phenomenon. For example, two models, *Drosophila melanogaster* (a fruit fly) and *Caenorhabditis elegans* (a nematode worm) have been used for decades to understand wiring in the brain, i.e., how its connections integrate sensing of and responding to the environment (see e.g., [5],[6]). The human brain has ∼85 billion neurons [7], *D. melanogaster* ∼100,000 [5], and *C. elegans* exactly 302 [6], with ∼10^14^, ∼10^7^, and ∼5,000 synapses, or connections, in each system, respectively. How does the complexity of neural integration compare with the interactions of the human microbiota? The number of bacteria in the average human is on the same order as the number of synapses in the human brain. However, one might argue that deciphering the communication among the bacterial microbiota, as well as between their communities and the host, offers an even more formidable challenge. Because this bacterial genetic diversity is so enormous, the number of bacterial genes “managing” the activities of the microbiota is conservatively dozens of times greater than that of the human genetic complement alone [8].

As with the alternative approaches used to understand brain complexity, some non-primate models for the study of symbiosis provide systems that lend themselves to experimental manipulation [9]. Those being explored fall into two broad categories: (1) germ-free or gnotobiotic, in-bred lines of vertebrates, particularly mouse and zebrafish; and, (2) certain simpler, natural invertebrate symbioses. In the latter, the microbial community is typically less diverse, with specific host tissues harboring between one and a few dozen phylotypes. For the study of symbiosis, each model has its strengths, with no one being either ideal or sufficient to provide a complete picture of how symbioses work. One binary association, the partnership between the Hawaiian bobtail squid, *Euprymna scolopes*, and the luminous bacterium *Vibrio fischeri* has offered a window into one area of interest: the colonization of animal epithelia by bacterial partners, perhaps the most common type of symbiosis in animals [10]. This video, produced by the “Science Nation” program of the National Science Foundation, provides some insight into our studies of this association under laboratory conditions. http://www.nsf.gov/news/special_reports/science_nation/glowingsquid.jsp

## A Most Agreeable Subject

**Figure 1:**
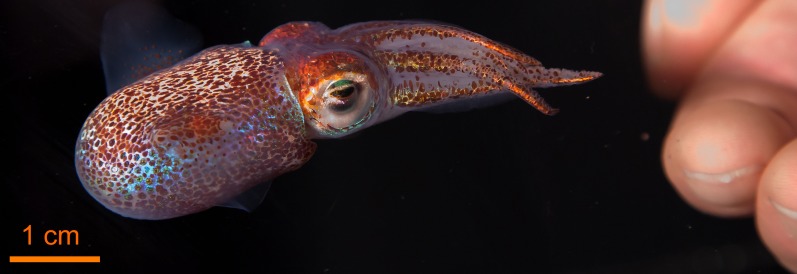
The adult host *Euprymna scolopes*, the Hawaiian bobtail squid. The human hand at the right offers an idea of the animal size.

The squid *E. scolopes* (Figure 1) live in the shallow reef flats of the Hawaiian archipelago. They are quiescent over the day, during which time they remain buried in the sand. Just after dusk, they emerge to forage in the water column for food. They can be collected in the evening with a dip net and flashlight. In addition to being used as a model for symbiosis, this species has been studied to provide a basic understanding of a wide array of biological phenomena [11], including development in cephalopods, the evolution of animal body plans, and the “design” of tissues that interact with light. The light produced by the bacterial symbionts is used in the host's behavior [12]. As one of many camouflage mechanisms, the squid emits ventral luminescence from a light-emitting organ deep in its body cavity. Although not well proven, biologists hypothesize that such behaviors in marine animals serve to make them less visible to predators looking up from below. The unique features of the system have enabled the study of symbiosis at all levels of the biological hierarchy, from the ecology and evolution of a symbiotic system to the underlying molecular mechanisms of partner interaction that lead to the establishment, development, and long-term persistence of the alliance.

The development of the squid-vibrio model of symbiosis began just over 25 years ago in the spring of 1988, as a collaborative effort between my laboratory and that of Edward (Ned) Ruby while we were on the faculty at the University of Southern California. At that time, intimate, coevolved symbioses between animals and microbes were thought to occur only rarely. The squid-vibrio system was attractive to us because it was a *natural* model that offered rare opportunities for the study of host-microbe alliances. The key characteristic it offered was then, and is still now, that rearing/culturing of host and symbiont separately under laboratory conditions is not physiologically compromising to either partner. Unlike most other associations, where symbionts provide essential nutrients or cofactors [13], the primary “currency” translocated from the bacterium to the host is light production; and, as a horizontally acquired symbiont, *V. fischeri*, a gram-negative bacterium that is a member of the Vibrionaceae in the γ-Proteobacteria, has an alternate niche as a culturable constituent of the bacterioplankton. The Vibrionaceae are a group of bacteria whose members often have broad physiological scope and multiple ecological niches. In addition to being constituents of the plankton, they are often associated with animals as enteric symbionts, pathogens, saprophytes, and mutualists [14]. In addition to nutrition not being the pivotal currency of the symbiosis, an equally compelling feature of the squid-vibrio association is its binary nature. In much the same way as it is easier to follow a conversation between two people than those of a group of interacting individuals, it is possible with a binary association to assign molecular and cellular signals and responses to a specific partner.

Over the early years of study, we explored the limits of the system as a model of symbiosis and were not disappointed. We found that maintaining a breeding colony in the lab of eight to ten mating pairs of adults yields ∼60,000 juveniles/year. In addition, Ned Ruby and his colleagues developed robust molecular genetic approaches in *V. fischeri*, which have provided valuable insight into critical features of both symbiont and host responses to their partnership. Although genetic manipulation is not yet available for the host, because this system is a binary one, modifying the symbiont genes has been used to define those bacterial products that modulate the host transcriptome. Finally, critical to the success of the work with the system is its tempo. As described more fully below, this highly specific symbiosis ensues within hours of the host hatching from the egg, early developmental events of the partners occur within a couple of days, and, from onset throughout the host's life, the symbiosis is on a profound daily rhythm.

Research of the squid-vibrio model has also been pushed along by its close tracking of tool development in imaging, genome sequencing, and functional genomics. By the mid to late 1990s, research on the system was well underway, with the ultrastructural relationships between the host tissues and its symbiont defined in both juvenile and adult animals [15],[16]. The application of confocal microscopy, which had just become broadly available, revealed important details of the 3-dimensional organization of the system, and allowed the visualization, in real time, of the events associated with symbiosis onset (see e.g., [17],[18]). This technology continues to be used to define the activities of individual genes, their products, and associated pathways of both partners through development of the association. In another arena, beginning about a dozen years ago, the “elephant in the room” was revealed to biologists in general, i.e., genomic methods became available to characterize the coevolved communities of bacteria that live in association with animals, plants, and other life forms. Technology enabled, the world of symbiosis research was to go from being the study of rare occurrences to a central theme in biology over the ensuing ten years. Genomic research opened doors for all symbiotic systems. For the squid-vibrio system in particular, full-genome sequences for symbiotic strains of *V. fischeri* are now available [19],[20]. For both partners, in the study of the transcriptome, we have moved from glass-slide microarrays [21]–[23] to RNA sequencing (RNAseq) [24]. In addition, proteomics has provided great insights into the biochemical environment of the host crypts that house the symbiont, as well as the symbiont cells' responses to this environment [25].

## Getting the Right Partner

**Figure 2:**
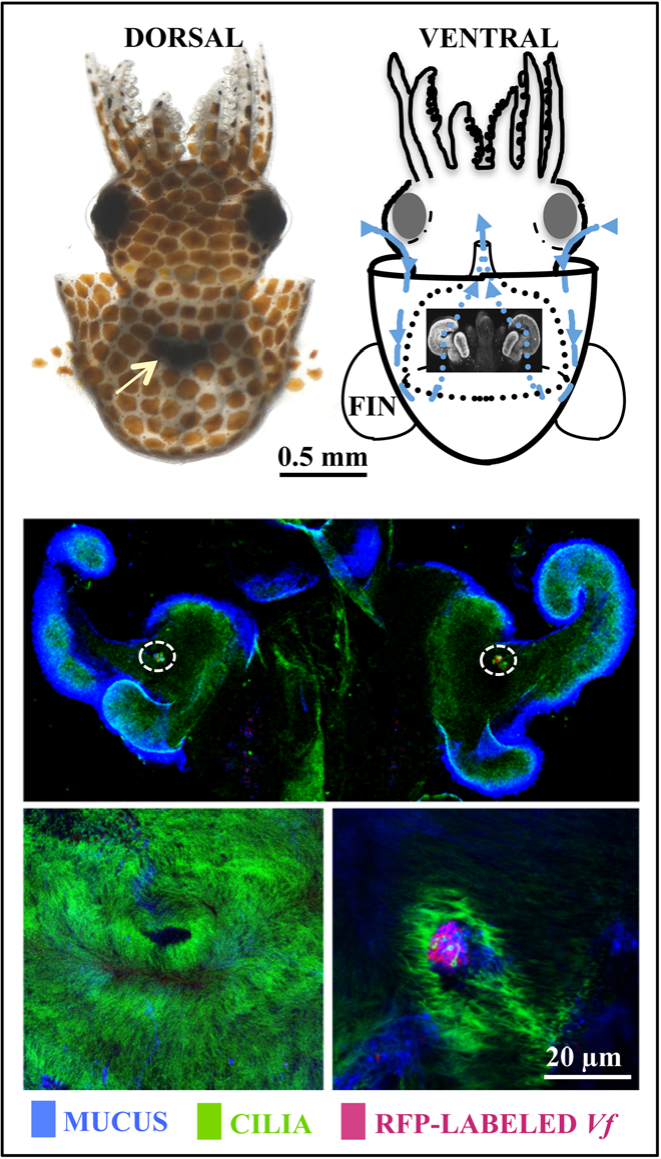
The recruitment of the symbiont *Vibrio fischeri* from the environment. Upper, left: the nascent light organ (black) can be seen through the body wall (white arrow) of the living juvenile animal; right: a diagram of a ventral view reveals the position of the light organ in the center of the mantle cavity. During ventilation, water that is rich with environmental bacteria passes over the organ (blue arrows). Middle, confocal microscopy of the juvenile organ reveals complex ciliated fields on either side that shed copious amounts of mucus. *V. fischeri* aggregates above pores on the surface (dashed circles). Lower, left: a dense field of cilia with a central pore, where *V. fischeri* cells will enter host tissues; right, aggregated *V. fischeri* cells entering host tissues. RFP, red fluorescent protein.

**Figure 3:**
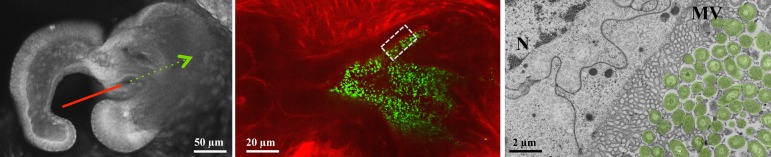
Colonization by the symbiont. Left, one half of the light organ illustrating the path of *V. fischeri* into the host tissues. Middle, confocal micrograph of green fluorescent protein (GFP)-labeled *V. fischeri* cells in a host crypt. Right, a transmission electron micrograph of *V. fischeri* associating with dense microvilli along the apical surfaces of host epithelia.

The squid-vibrio model symbiosis has been used to study all phases of a horizontally acquired association: onset, development, and maintenance [10]. The take-home message from the 25 years of study is that this trajectory, even in a seemingly simple binary association, can be highly complex. Shortly after hatching, the squid sheds mucus from a superficial ciliated epithelium (Figure 2). It is along this tissue that *V. fischeri* cells are harvested from the environment. After a few hours of aggregating, these cells move as a group into pores on the surface of the tissues, travel down ducts, and proliferate into dense populations in deep crypts (Figure 3). Several surprises about these initial events arose from analysis of the onset using confocal microscopy and bacterial genetics.

The first surprise was the discovery of when and where specificity is determined during initiation of the partnership. Because no other bacteria colonize the host crypts, our initial hypothesis was that the specificity is determined in the crypts, not on the surface of the organ, which is exposed to abundant (∼10^6^/ml of seawater) non-specific environmental bacteria. We were wrong. A series of studies showed that when *V. fischeri* cells are present, they associate with the host along the superficial epithelium, a process that occurs in two steps: attachment to the cilia by an unknown mechanism [26], followed by aggregation of the harvested population, which requires a symbiont-produced exopolysaccharide [27]. In the absence of *V. fischeri*, other Gram-negative, but not Gram-positive, bacteria will aggregate; however, when would-be symbiont cells are present, they exhibit a competitive dominance and, after ∼3 h are the only cells present in the aggregates. How does this exclusion occur? Transcriptomic studies of the tissues have revealed that the host recognizes and responds to the presence of the symbiont [24]. While yet to be definitively proven, data from these and other studies suggest that the host responses change the game, balancing the biochemical environment in favor of the symbiont. Further, these studies demonstrated that the bacteria induce a change in gene expression of a host chitinase that primes the bacteria to chemotax up a chitobiose gradient into host tissues. Thus, the studies of the initiation processes have demonstrated that elimination of other bacterial species requires a complex host-symbiont dialogue across the ciliated surface epithelium. After entering the pores, the harvested *V. fischeri* cells travel toward the crypts, which occur beyond a bottleneck, or constriction in the epithelial tissue. Another unexpected finding was that, no matter how large the group of migrating cells, only one or two make it beyond the bottleneck, where they begin to divide, eventually filling the crypt spaces [28]. Taken together, the data have shown that, over the distance of a couple of hundred microns, the system orchestrates a dramatic “winnowing” from thousands of environmental bacterial species interacting with the surface of the organ to one to two strains of *V. fischeri* in the deep crypts.

## Developing a Lasting Relationship

Historically, much of our effort has centered on understanding development of the association. Our earliest peer-reviewed publication on the system presented the first report of bacteria-induced development in animal tissues, similar to induction of nodule formation in leguminous plants by nitrogen-fixing symbionts [29]. Once in the crypts, *V. fischeri* cells induce loss of the superficial ciliated fields that facilitate their colonization. A focused effort on this process demonstrated that cell-surface molecules of the symbiont, specifically the monomer of cell-wall peptidoglycan and the lipid A component of lipopolysaccharide (LPS) of the outer membrane, work synergistically to induce regression of the ciliated field by apoptosis [30]. The structures of these “microbe-associated molecular patterns,” or MAMPs, are largely conserved throughout evolution, but specificity of the response is achieved by their presentation to the crypt epithelium where only *V. fischeri* can reside. This finding was unexpected as these molecules had been thus far only associated with the activity of bacterial pathogens, although concomitant studies had shown that interactions with LPS in the mammalian gut are similarly essential for gut homeostasis [31]. Further, it had long been known that some feature of Gram-negative bacteria induces maturation of the gut-associated lymphoid tissue (GALT) in mammals. A few years after the publication of MAMP-induced development in the squid, Gerard Eberl's group at the Pasteur Institute reported that GALT maturation is induced by the peptidoglycan monomer, one of the exact same molecules that actively induce development in the squid [32]. In hindsight, perhaps it is not surprising that responses to these conserved molecules are widespread, as animals have been interacting with them since the earliest days of the diversification of all animal body plans.

MAMPs do not work alone. Normal development also requires that the symbionts do their “job,” i.e., produce luminescence. *V. fischeri* cells defective in light production (Δlux) are significantly delayed in symbiont-induced developmental phenotypes [33]. Such “cheating” mutants are sanctioned by the host; they are not retained in symbiosis, being eliminated from the crypts within a few days of colonization. How does the light organ sense symbiont light production? Studies of light-organ tissues revealed expression of all of the components of the eye's visual transduction cascade, including rhodopsin [34], the visual pigment that perceives light. Like the eye, the light organ is physiologically responsive to light, a behavior that can be detected by electroretinograms, which measure electrical responses to exogenous light cues. Further, transcriptomic studies of host tissues colonized by Δlux demonstrated that light is critical in induction of normal host gene expression, most notably the expression of the host's receptors to symbiont MAMPs, or pattern-recognition receptors (PRRs), the elements critical for developmental induction [35]. Thus, the light organ is an “inner eye,” which can produce bioluminescence as well as perceive it.

## They've Got Rhythm

Once the symbiont colonizes the tissues, how is it maintained in balance throughout the life of the host? Two early studies demonstrated that the squid-vibrio system is on a daily rhythm. The per-cell luminescence of the bacteria is brightest in the evening, when the squid is out foraging [36], and ∼90% of the symbiont population is expelled from the light organ each day at dawn [37]. When the technology became available, we explored the host and symbiont transcriptome over the day-night cycle to discover the dialogue between the partners underlying these daily rhythms [38]. The resulting data led to the finding that the host crypt epithelium undergoes dramatic remodeling over the day-night cycle. These diel changes in host tissues appear to reflect variations in the food source provided to the bacterial symbionts. In response, bacterial gene expression vacillates from anaerobic respiration to anaerobic fermentation, a switch that would dramatically alter the pH environment of the crypts. These data demonstrate that maintenance of the squid-vibrio symbiosis is a dynamic process with a profound daily rhythm.

The presence in the light-organ's transcriptomic database of genes that encode cryptochromes (Cry), blue-light receptive proteins controlling circadian rhythms, suggested possible circadian control of the observed patterns in the symbiosis [39]. We identified two different Cry isoforms in the host. The gene encoding one cycles in the head with a pattern similar to that described in other animals, where the response is to diel environmental light cues; in contrast, the other *cry* gene has higher expression in the light organ and responds to the cycling of symbiont luminescence. The cycling of the light organ *cry* gene requires the presence of the symbiont, and the symbiont must be luminous, i.e., Δlux strains do not induce cycling of the light-organ *cry* gene. Further, similar to development, to obtain normal *cry* cycling in the light organ requires not only the presence of the bacteria, but also their presentation of MAMPs, which appear to synergize with symbiont luminescence.

Although this work in the squid-vibrio system was the first to report bacteria inducing circadian rhythms in a host animal, such responses are likely to be a widespread phenomenon. For example, circadian rhythms in gene expression occur in both the gut epithelia [40] and mucosal immune systems [41] of mammals. The extent to which the third component in that environment, the large microbial populations, are affected by and/or entrain these rhythms remains to be determined. However, a recent study of the mammalian gut has demonstrated that circadian rhythms are essential for the MAMPs-mediated homeostasis of the gut [42]. We predict that circadian rhythms will be central to the form and function of host-microbe interactions in many animals, an impact that will have broad implications for research in the field. For example, the future design of germ-free mouse facilities for researchers investigating the normal microbiota should consider environmental conditions to entrain the animals on a day-night cycle.

## Horizons

Twenty-five years of research on this simple system have demonstrated that we have only just begun to understand the broader outlines of the squid-vibrio symbiosis. New opportunities, made available through continuing development of technology, will bring the system to a new level of exploration. Currently, efforts are underway to develop single-cell transcriptomics in the symbiont, an advance that promises to shed light on the molecular underpinnings of habitat transition from environmental to symbiotic niches. In addition, the continuing decline in the cost of nucleic-acid sequencing presents new opportunities for the study of both host and symbiont. The sequencing of the squid genome, which is ∼107% the size of the human genome (Animal Genome Size Database; www.genomesize.com), is now underway at the sequencing facilities of Washington University at St. Louis, as a collaboration between my laboratory and that of Spencer Nyholm (U Connecticut). These data will afford the opportunity to study host-gene regulation both during the onset and throughout the maintenance of symbiosis. Determination of full-genome sequences of dozens of *V. fischeri* strains is also ongoing. These data will provide insight into how mixed populations, common in both binary and consortial associations, are structured within a symbiosis. Further these new data will allow us to define the pangenome of the symbiont, which will in turn help define the core genome critical for a horizontally transmitted symbiont. We are also continuing to explore methods for bringing genetics into the host animal. Finally, research directions for the near future include expansion into approaches using physics, engineering, and mathematical modeling to understand the capture of bacteria by ciliated epithelial surfaces. Our continuing goal is to define conserved processes governing symbiotic associations, with the hope that we will provide fruitful directions for the study of more complex systems.
